# Landscape and mosquito community impact the avian *Plasmodium* infection in *Culex pipiens*

**DOI:** 10.1016/j.isci.2024.109194

**Published:** 2024-02-10

**Authors:** Martina Ferraguti, Josué Martínez-de la Puente, Santiago Ruiz, Ramón C. Soriguer, Jordi Figuerola

**Affiliations:** 1 Departamento de Biología de la Conservación y Cambio Global, Estación Biológica de Doñana (EBD), CSIC, C/Américo Vespucio, 26, 41092 Seville, Spain; 2 Department of Parasitology, University of Granada (UGR), Granada, Spain; 3 Servicio de Control de Mosquitos, Diputación de Huelva, Huelva, Spain; 4 CIBER de Epidemiología y Salud Pública (CIBERESP), Madrid, Spain

**Keywords:** Ecology, Microbiology, Microbiology parasite

## Abstract

Avian malaria parasites provide an important model for studying host-pathogen interactions, yet understanding their dynamics in vectors under natural conditions is limited. We investigated the effect of vector abundance, species richness and diversity, and habitat characteristics on avian *Plasmodium* prevalence and lineage richness in *Culex pipiens* across 45 urban, natural, and rural localities in southern Spain*.* Analyzing 16,574 mosquitoes grouped in 768 mosquito pools, 32.7% exhibited parasite presence. 13 different *Plasmodium* lineages were identified, with the lineage SYAT05 being the most commonly found. Parasite prevalence positively correlated with the distance to saltmarshes and rivers, but negatively with the distance to total water source. Parasite lineage diversity was higher in natural than in rural areas and positively correlated with mosquito species richness. These results emphasize the complex dynamics of avian *Plasmodium* in the wild, with habitat characteristics and vector community driving the parasite transmission by mosquito vectors.

## Highlights

• *Plasmodium* prevalence in mosquitoes was higher farther from saltmarshes and rivers
• *Plasmodium* prevalence showed a negative correlation with the mosquito abundance
• *Plasmodium* lineage richness in mosquitoes was lower in rural than in natural areas
• *Plasmodium* lineage richness increased with mosquito species richness and diversity

## Graphical abstract

**Figure:**
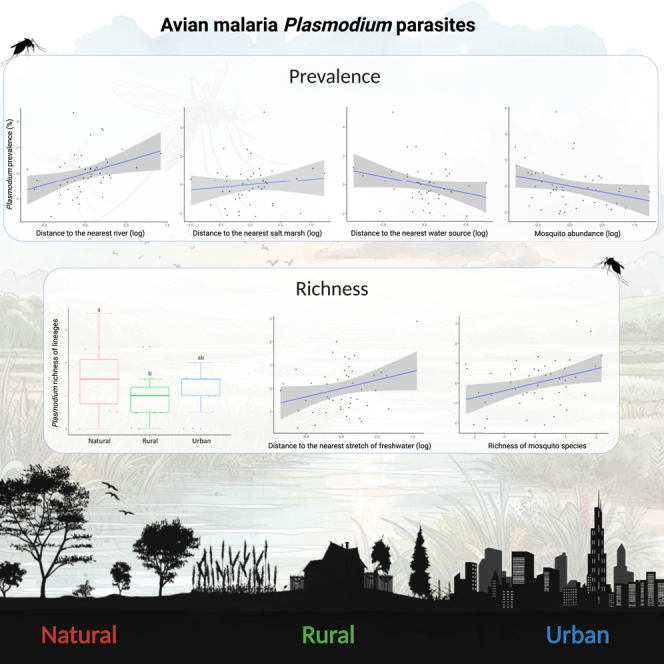

## Introduction

The transmission dynamics of vector-borne pathogens with complex life cycles are significantly influenced by environmental characteristics. This is the case of avian malaria parasites, which rely on competent mosquito vectors for efficient transmission from infected individuals to new hosts.^1^ Avian malaria parasites of the genus *Plasmodium*, a widespread haemosporidian, infect a variety of bird species across all continents except Antarctica.^1^ Among the various mosquito species, those belonging to the *Culex* genus play a crucial role in the transmission of avian malaria parasites, with *Culex pipiens* repeatedly identified as a major vector.^2^^,^^3^ This species has a wide distribution inhabiting the Holarctic region and is found extensively across Europe.^4^ *Culex pipiens* has a relatively opportunistic feeding behavior with birds representing a large, but variable, proportion of its diet.^5^^,^^6^ In addition, infective forms of avian *Plasmodium* have been found in the salivary glands of this species,^2^ and parasite DNA has been detected in its saliva.^3^ Numerous studies have reported the frequent presence of *Plasmodium* parasites in pools of *Cx. pipiens* captured in the wild,^6^^,^^7^^,^^8^^,^^9^ further supporting the relevance of this species in the transmission of avian malaria under natural conditions.

Different factors, including both abiotic and biotic variables,^10^^,^^11^ influence the susceptibility of hosts to parasite infections which result in differential infection patterns among bird populations.^12^ Environmental conditions can determine the dynamics of parasite transmission,^13^^,^^14^ and anthropogenic activities such as urbanization and deforestation affect the infection patterns of vector-borne pathogens, including avian malaria.^14^ Habitat affects the prevalence of haemosporidians in wild birds^15^ as well as the temporal variability in the prevalence of parasite infections.^16^ These effects are probably mediated by the impact of landscape characteristics on vector populations,^17^^,^^18^ but the direct association between these variables has been rarely explored. Previous studies have highlighted the importance of factors such as the proximity to water resources on mosquito populations^19^^,^^20^^,^^21^ and thus, directly and/or indirectly, affecting the pathogen transmission risk.^22^^,^^23^ Water availability was shown to be one of the most important predictors of avian malaria incidence in wild birds,^24^^,^^25^ with a higher prevalence of infection in birds breeding or captured near water sources.^26^^,^^27^^,^^28^ Small water bodies provide suitable habitats for mosquito breeding, supporting higher numbers of insect vectors.^11^ Consequently, the increased abundance of vectors may elevate the risk of vector-borne parasite infections in wild birds.^29^

Here we explore the association between landscape and avian *Plasmodium* prevalence in *Cx. pipiens* mosquitoes captured at 45 localities, to better understand the sources of variation in the prevalence of avian *Plasmodium* parasites in its main vector. We aim to assess the importance of habitat and water source parameters on the prevalence and richness of avian *Plasmodium* lineages in *Cx. pipiens*. We predict that water proximity, as related to a higher number of potential breeding areas, may favor parasite infections in vectors as previously shown in birds. Identifying the factors that determine the interactions between *Cx. pipiens* and avian malaria parasites, including the landscape characteristics, is essential for understanding the transmission dynamics of this vector-borne disease.

## Results

### Parasite identification in mosquitoes

Overall, 16,574 *Cx. pipiens* females grouped in 768 pools were analyzed. Avian *Plasmodium* was amplified in 251 of the mosquito pools (32.7%), including 13 mixed infections indicated by the presence of double peaks in the chromatogram. In addition, due to the low quality of sequences, the identification of amplicons from two positive mosquito pools was limited to the genus level.

**Figure 1:**
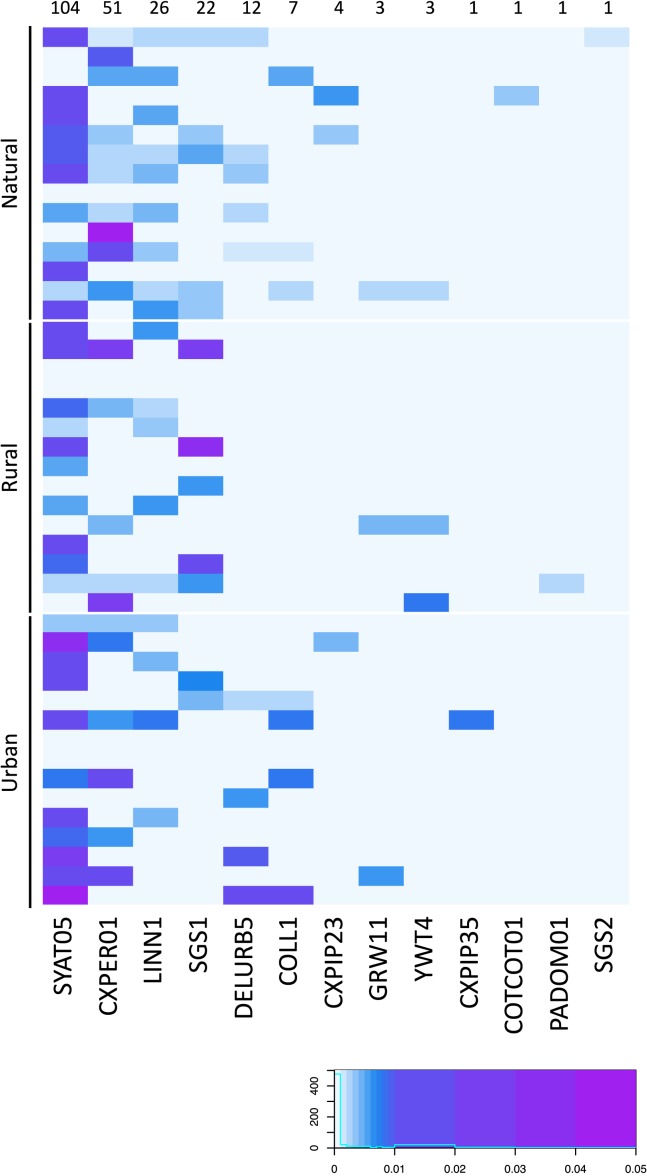
Heatmap representing the prevalence of *Plasmodium* lineages detected in mosquito populations across 45 sampling locations The lineages were labeled according to MalAvi nomenclature and sorted in decreasing order according to the number of infected mosquito pools found, as indicated by the numbers above each column.

Overall, we identified the presence of 13 distinct *Plasmodium* lineages (Figure 1; Table S1). The most common *Plasmodium* lineage detected in mosquito pools was SYAT05 (n = 104), followed by CXPER01 (n = 51), LINN1 (n = 26), SGS1 (n = 22), and DELURB5 (n = 12). The remaining lineages were observed in seven or fewer mosquito pools. Notably, four of these lineages corresponded to known morphospecies: *P. matutinum* (LINN1), *P. vaughani* (SYAT05), and *P. relictum* (SGS1 and GRW11). Additionally, one lineage, CXPIP35, was described for the first time in this study (see Table S1). Additionally, we identified three other *Haemoproteus* lineages in mosquitoes (two *Haemoproteus* sp. PAHIS1 and one *Haemoproteus passeris* PADOM05). However, since *Haemoproteus* parasites are not transmitted by mosquitoes,^1^ they were not included in the following analyses.

### Effects of habitat, water sources, and mosquito community composition on *Plasmodium* infections

**Figure 2:**
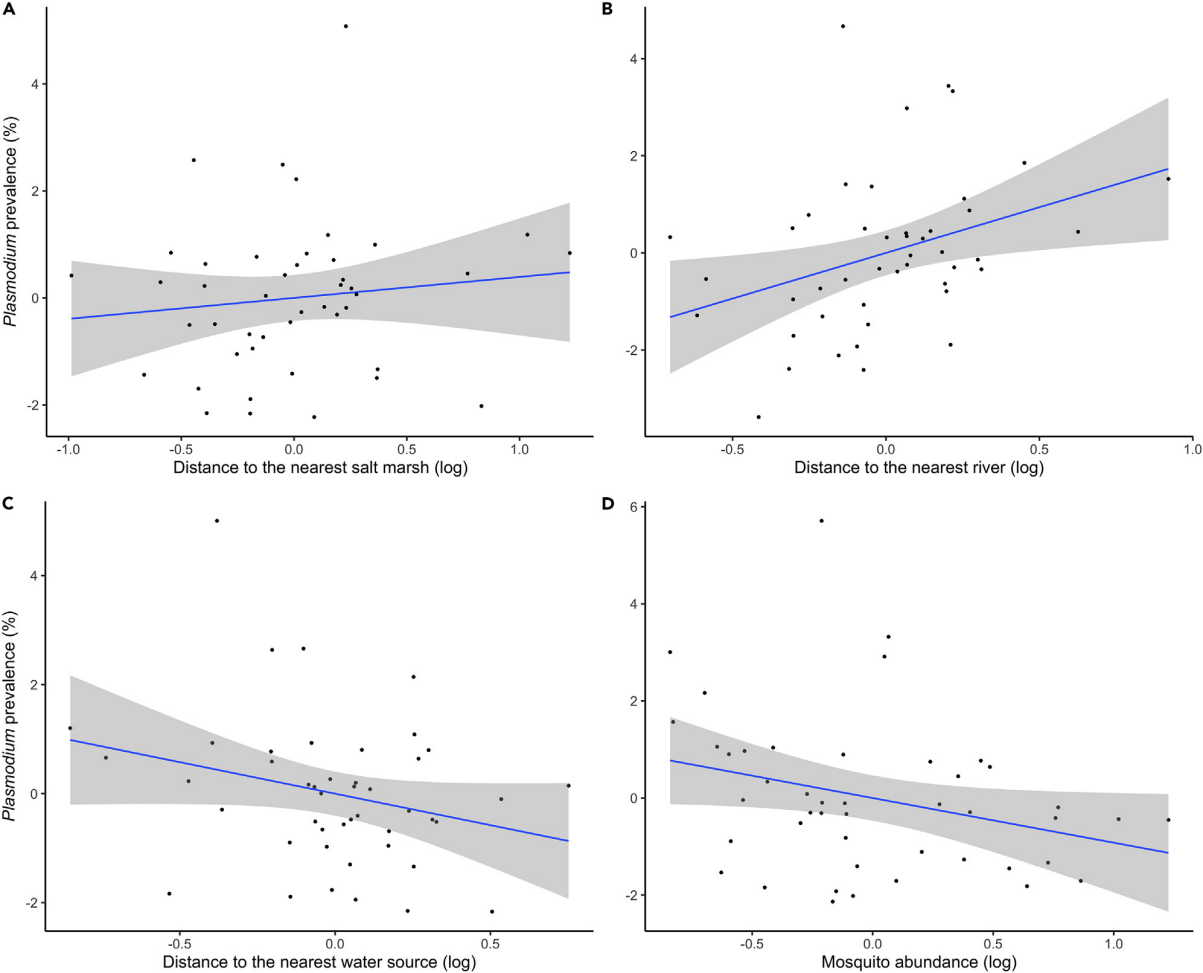
Relationships between *Plasmodium* prevalence in *Culex pipiens* and water and mosquito variables Leverage plot displaying the residuals of the model, accounting for the associations between *Plasmodium* prevalence in *Cx. pipiens* at the 45 localities and the distance in meters to the nearest (A) salt marshes, (B) rivers, (C) water sources, and (D) total mosquito abundance. The 95% confidence level interval is shown in gray.

The mean *Plasmodium* prevalence in the study area was 2.42%, ranging from 0% to 10.30% across localities (Table 1). We observed no evidence of collinearity among the variables included in the models. *Plasmodium* prevalence showed a positive correlation with the distance to salt marshes (Figure 2A) and rivers (Figure 2B), while it exhibited a negative correlation with the distance to any water source (Figure 2C) and the total mosquito abundance (Figures 2D; Table 2). Additionally, no significant differences were observed in parasite prevalence among various habitat types. This was also true for the prevalence of the predominant lineages—SYAT05, CXPER01, LINN1, and SGS1—in mosquitoes (all p > 0.05 in linear mixed-effects models [LMMs]).

**Table 1:** Geographic location, number of *Culex pipiens* pools molecularly tested for the presence of avian *Plasmodium*, number of females, *Plasmodium* prevalence and the richness of lineages (measured from a rarefaction curve) at each sampling locality

Province	Category	Latitude	Longitude	Nº Pools (Nº individuals)	*Plasmodium* prevalence	Lower 95% CL	Upper 95% CL	*Plasmodium* lineage richness
Cadiz	Natural	36.71483	−6.43458	15 (262)	0.028	0.010	0.062	4.848
Cadiz	Rural	36.71260	−6.41175	12 (173)	0.016	0.003	0.052	3.000
Cadiz	Urban	36.73072	−6.42998	14 (181)	0.000	0.000	0.000	3.000
Cadiz	Natural	36.61384	−6.05702	16 (466)	0.062	0.027	0.138	2.000
Cadiz	Rural	36.62129	−6.08174	10 (137)	0.037	0.009	0.104	1.000
Cadiz	Urban	36.68165	−6.12553	18 (196)	0.006	0.000	0.025	4.000
Cadiz	Natural	36.57122	−6.21524	13 (233)	0.018	0.004	0.047	3.000
Cadiz	Rural	36.61767	−6.21791	16 (241)	0.004	0.000	0.019	3.000
Cadiz	Urban	36.58956	−6.23852	19 (297)	0.019	0.006	0.044	3.000
Cadiz	Natural	36.63576	−6.39296	19 (430)	0.059	0.027	0.121	3.000
Cadiz	Rural	36.66221	−6.39714	18 (202)	0.005	0.000	0.022	2.000
Cadiz	Urban	36.67188	−6.40609	20 (286)	0.055	0.027	0.098	2.000
Cadiz	Natural	36.84686	−6.31754	25 (830)	0.021	0.011	0.036	0.000
Cadiz	Rural	36.82013	−6.32531	18 (306)	0.012	0.003	0.031	4.000
Cadiz	Urban	36.77655	−6.35369	17 (122)	0.029	0.007	0.074	3.000
Huelva	Natural	37.42428	−6.81974	22 (764)	0.026	0.014	0.043	0.000
Huelva	Rural	37.42753	−6.84388	12 (86)	0.051	0.016	0.114	0.000
Huelva	Urban	37.42352	−6.79923	14 (67)	0.103	0.037	0.215	5.000
Huelva	Natural	36.98077	−6.48341	15 (364)	0.026	0.010	0.055	3.000
Huelva	Rural	36.98870	−6.44304	15 (103)	0.011	0.004	0.026	2.000
Huelva	Urban	37.12745	−6.47998	14 (317)	0.023	0.008	0.050	2.000
Huelva	Natural	37.36417	−6.97879	46 (1819)	0.023	0.015	0.033	1.000
Huelva	Rural	37.38273	−6.98785	18 (436)	0.013	0.005	0.028	1.000
Huelva	Urban	37.37025	−6.96375	15 (139)	0.030	0.012	0.082	2.000
Huelva	Natural	37.25354	−6.96847	19 (430)	0.025	0.011	0.050	3.000
Huelva	Rural	37.27903	−6.90975	22 (607)	0.022	0.013	0.035	1.000
Huelva	Urban	37.2609	−6.95989	18 (246)	0.000	0.000	0.000	4.000
Huelva	Natural	37.33844	−6.72314	23 (702)	0.031	0.017	0.053	0.000
Huelva	Rural	37.33132	−6.76887	11 (179)	0.024	0.006	0.064	0.000
Huelva	Urban	37.31204	−6.84878	19 (534)	0.009	0.003	0.020	3.000
Seville	Natural	37.23839	−6.12955	6 (123)	0.018	0.006	0.039	4.000
Seville	Rural	37.21710	−6.18575	4 (12)	0.000	0.000	0.000	1.000
Seville	Urban	37.28360	−6.06549	15 (132)	0.034	0.011	0.078	4.411
Seville	Natural	37.35825	−5.90366	21 (656)	0.014	0.006	0.027	1.000
Seville	Rural	37.24940	−6.02383	24 (788)	0.005	0.002	0.013	2.000
Seville	Urban	37.35341	−5.93608	22 (400)	0.021	0.008	0.043	2.000
Seville	Natural	37.36375	−5.22964	34 (1291)	0.031	0.019	0.047	2.000
Seville	Rural	37.35001	−5.22325	10 (50)	0.063	0.016	0.155	2.000
Seville	Urban	37.34943	−5.22919	12 (200)	0.034	0.012	0.073	1.000
Seville	Natural	37.42302	−5.99415	14 (145)	0.015	0.003	0.046	7.000
Seville	Rural	37.34601	−5.94310	28 (940)	0.015	0.008	0.026	3.000
Seville	Urban	37.35843	−5.97869	17 (170)	0.038	0.011	0.091	3.000
Seville	Natural	37.01909	−6.16245	5 (18)	0.000	0.000	0.000	3.000
Seville	Rural	37.03033	−6.19003	4 (96)	0.000	0.000	0.000	5.000
Seville	Urban	37.13163	−6.16317	19 (398)	0.014	0.005	0.030	2.000

Parasite richness was estimated from a rarefaction curve.

**Table 2:** Relationships between *Plasmodium* prevalence and the richness of lineages (measured from a rarefaction curve) in *Culex pipiens* per locality (N = 45), and different environmental variables, as estimated from LMMs

Independent variable	*Plasmodium* prevalence					*Plasmodium* richness				
	*Estimate (±S.E.)*	*χ*2	df.	*t*	*p*	*Estimate (±S.E.)*	*χ*2	df.	*t*	*p*
Intercept	0.043 (0.022)	3.745	1	1.935	0.073	−0.877 (1.397)		1	−0.628	0.534
Mosquito abundance	**−0.004 (0.002)**	**4.283**	1	**−2.070**	**0.045**	0.026 (0.405)	0.004	1	0.064	0.949
Richness of mosquitoes	0.001 (0.001)	0.037	1	0.192	0.849	**0.316 (0.138)**	**5.216**	1	**2.284**	**0.028**
Diversity of mosquitoes	0.005 (0.008)	0.439	1	0.663	0.511	0.918 (1.341)	0.468	1	0.685	0.498
Habitat: natural	0.000^a^	0.920	2			0.000^a^	**7.977**	**2**		
Habitat: rural	−0.002 (0.003)			−0.834	0.411	**−1.255 (0.462)**			**−2.715**	**0.011**
Habitat: urban	−0.001 (0.003)			−0.085	0.933	−0.462 (0.554)			−0.834	0.410
Distance to any nearest water source	**−0.005 (0.002)**	**5.355**	**1**	**−2.314**	**0.026**	0.254 (0.316)	0.645	1	0.803	0.427
Distance to the nearest salt marsh patch	**0.003 (0.001)**	**5.213**	**1**	**2.283**	**0.031**	0.275 (0.212)	1.680	1	1.296	0.202
Distance to the nearest water reservoir	0.010 (0.005)	3.505	1	−1.872	0.079	−0.161 (1.095)	0.021	1	−0.147	0.885
Distance to the nearest stretch of freshwater	0.003 (0.002)	2.490	1	1.578	0.123	**0.577 (0.283)**	**4.146**	1	**2.036**	**0.049**
Distance to the nearest river course	**0.006 (0.003)**	**4.323**	**1**	**2.079**	**0.044**	0.278 (0.432)	0.414	1	0.643	0.524
***R***^***2***^**(%)**	31.2 (39.6)					23.8 (46.2)				

Significant relationships (p ≤ 0.05) are highlighted in bold; marginal (and conditional) R2 variance is shown for each model. For variables not included in the final model, the significance when added to the final model is given.

a Reference category.

**Figure 3:**
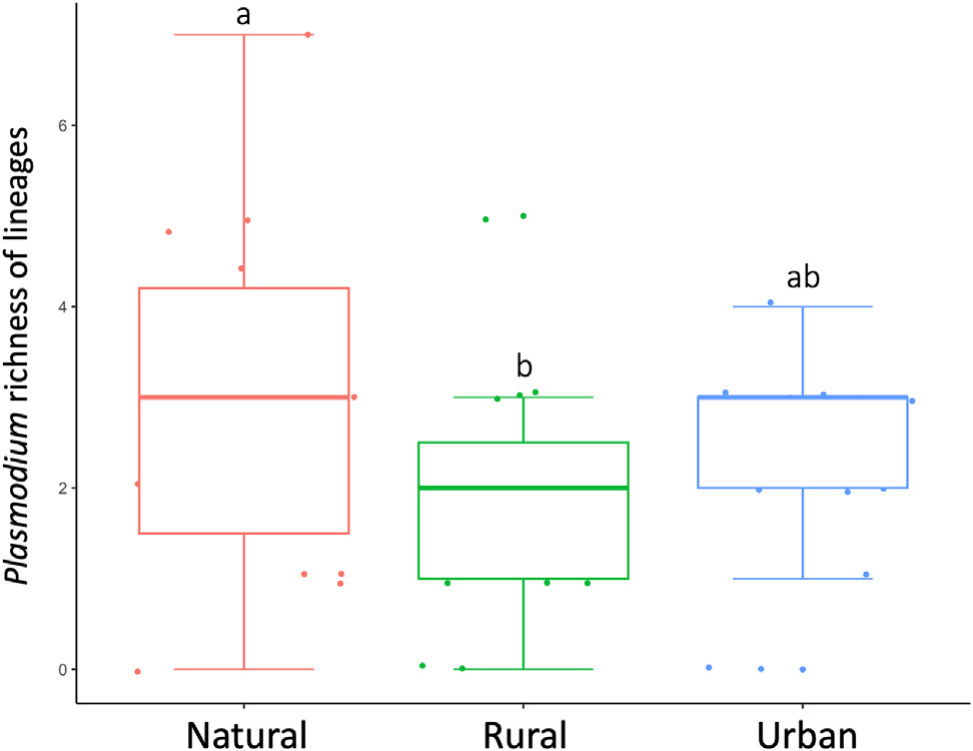
Richness of *Plasmodium* lineages in *Culex pipiens* as estimated from a rarefaction curve Median values, error bars, and data points (filled circles) are shown. Habitat categories with statistically significant differences (p ≤ 0.05) are marked with different letters.

**Figure 4:**
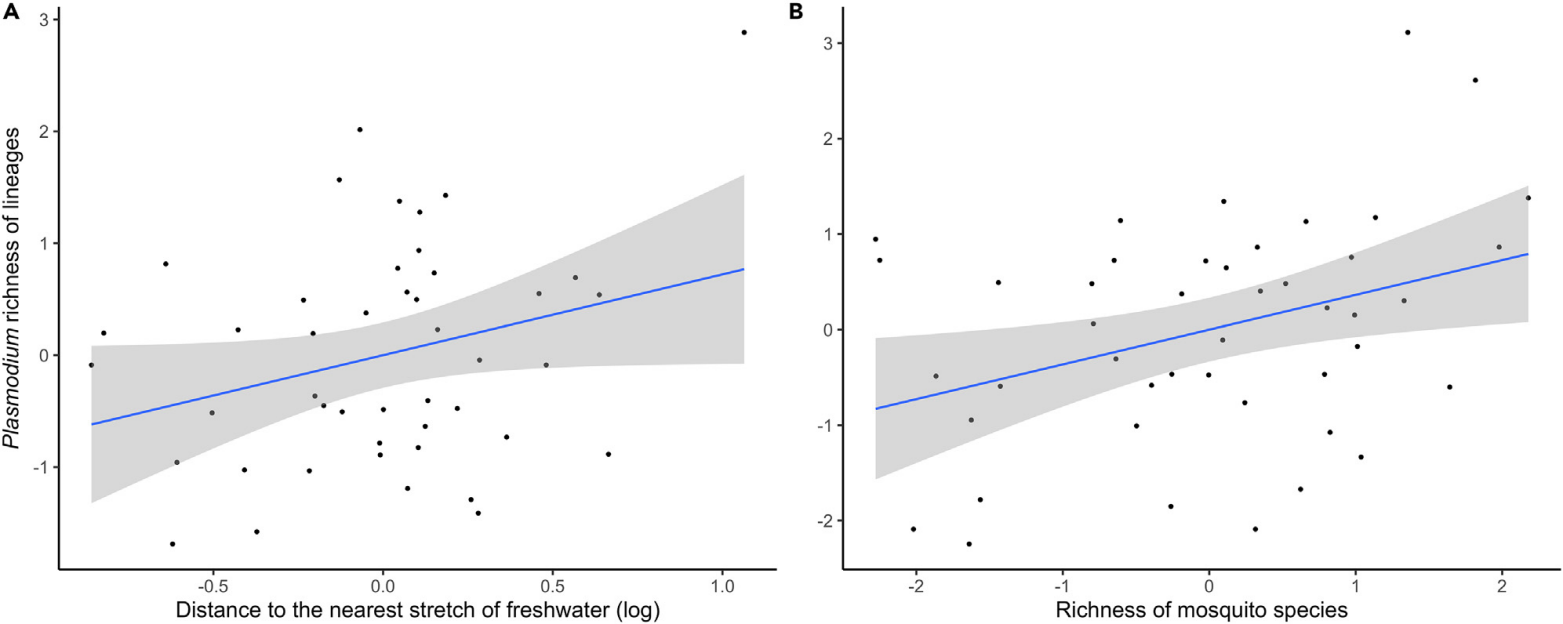
Relationships between *Plasmodium* richness in *Culex pipiens* and water and mosquito variables Leverage plot displaying the residuals of the model, accounting for the associations between *Plasmodium* richness of lineages in *Cx. pipiens* and (A) the distance in meters to the nearest stretch of freshwater and (B) the richness of mosquito species at the 45 localities studied. The 95% confidence level interval is shown in gray.

The mean richness of *Plasmodium* lineages was 2.45, ranging from 0 to 7 lineages per locality (Table 1). Habitat type was significantly associated to the richness of *Plasmodium* lineages (ANOVA: χ^2^ = 7.649, df. = 2, p = 0.022; Figure 3), with lower richness of lineages in rural than in natural areas (Tukey *post hoc* test: estimate = −1.216, S.E. = 0.489, *z* = −2.488, p = 0.033). The *Plasmodium* lineage richness in *Cx. pipiens* was positively related to the distance to the nearest stretch of freshwater (Figure 4A) and to the richness of different mosquito species (range from 2 to 10 species per locality) found in the studied areas (Figure 4B; Table 2).

## Discussion

Traditionally, the study of avian malaria parasites has focused on investigating the relationships between bird hosts and their parasites, while largely overlooking the role of the vectors. By providing new insights into the local circulation of different *Plasmodium* lineages in mosquitoes from southern Spain, our study expands the existing knowledge of the environmental drivers of parasite infections in mosquito vectors.

Environmental factors play a prominent role in driving the transmission dynamics of vector-borne pathogens. This is the case of avian malaria parasites, where the role of landscape has been supported in different vertebrate species.^11^^,^^26^^,^^30^^,^^31^ However, vectors may be also important determinants of the patterns of *Plasmodium* infections in bird hosts. For instance, parasite prevalence in house sparrows was largely determined by the composition of vectors in the area, with no apparent correlation with habitat categories.^11^ Similarly, seroprevalence of antibodies against the mosquito-borne West Nile virus (WNV) in this species was found to be positively correlated with the abundance of their primary mosquito vector, specifically *Cx. perexiguus*.^22^ However, notable differences may exist between patterns found in birds and mosquitoes, as the high mobility of birds (e.g., through natal dispersal or long-distance migration) introduces an additional layer of complexity in order to identify these ecological patterns.^32^ Migratory birds can introduce parasites to habitats where they were previously not found^33^ and thus shape the world-wide distributions of parasites.^34^ This recognition is crucial in understanding the complex interplay between habitats, vectors, and parasite dynamics which could be useful for conducting effective strategies for disease control and prevention.

### Parasite infection patterns in mosquitoes

We screened avian malaria parasites in *Cx. pipiens* mosquitoes, a key vector species in Europe, revealing an average *Plasmodium* prevalence of 2.42% in the area. This prevalence value is within the range previously found in other European areas. For instance, previous studies on this mosquito species in the Iberian Peninsula reported *Plasmodium* prevalence rates ranging from 0.04% up to 8.75%.^6^^,^^7^^,^^9^^,^^35^ In addition, studies in other countries have found avian *Plasmodium* prevalence in *Cx. pipiens* mosquitoes of 6.6% and 15.4% in Switzerland,^8^^,^^36^ and 5.9% in areas spanning Hungary and Serbia.^37^

In addition, we found 13 different *Plasmodium* lineages harbored by *Cx. pipiens*, further supporting the significant role of this species as a potential vector of a wide range of *Plasmodium* lineages. Notably, certain of these lineages were detected across various environmental settings, including natural, rural, and urban environments (Figure 1). For instance, the SYAT05, CXPER01, LINN1, and SGS1 lineages were found in all three habitats, underscoring their adaptability to diverse environments. Conversely, lineages such as DELUB5, COLL1, and CXPIP23 were identified in both urban and natural areas but not in rural locations. On the other extreme, certain specialist lineages were exclusive to specific localities. For instance, SGS2 and COTCOT01 were solely found in one natural locality each, PADOM01 was unique to a rural area, and a novel lineage, CXPIP35, was first described in an urban environment during this study (Figure 1).

The development of parasites in mosquito species is strongly determined by both genetic or biotic factors (e.g., insect microbiota) which may vary between insect species and individuals.^38^ Although the molecular identification of parasites in mosquitoes captured in the wild is not enough to demonstrate vector competence,^39^^,^^40^ the capacity of *Cx. pipiens* for the transmission of different avian *Plasmodium* lineages has been repeatedly supported.^2^^,^^3^ This also applies for the different ecotypes of the *Cx. pipiens* species, including *Cx. pipiens pipiens*, *Cx. pipiens molestus*, and their hybrids. We did not account for the intraspecific variation (e.g., genetic haplotypes) of mosquito vectors in the area which could partially affect their interactions with parasites.^41^ Although the different *Cx. pipiens* ecotypes are known to occur in the study area,^42^ all of them may be involved in the local transmission of avian *Plasmodium*.^35^^,^^42^ Overall, 149 different avian *Plasmodium* lineages have been molecularly recorded in *Cx. pipiens* (Vector Data Table in MalAvi, version 2.5.8) supporting the role of this species as a generalist vector of avian malaria parasites. Interestingly, the lineage SYAT05 showed, by far, the highest prevalence in mosquitoes across all habitats (Figure 1) representing 104 of the 236 positive mosquito pools. The lineage SYAT05 has been recorded in 37 different host species of 17 bird families and used to be a common parasite lineage also found in seven species of vectors^43^^,^^44^^,^^45^ (Vector Data Table in MalAvi, version 2.5.8). Blackbirds *Turdus merula* are common hosts of *Plasmodium* SYAT05^46^ and preferred blood meal sources for *Cx. pipiens*,^47^ likely resulting in a high prevalence of this parasite lineage in mosquitoes where this host species is present.^43^^,^^44^^,^^45^

### Impact of habitat and water source on avian malaria parasites in mosquitoes

*Culex pipiens* is a widely distributed mosquito that inhabits the Holarctic region and is found extensively across Europe.^48^ This species has a remarkable ability to adapt to various water sources, occupying a wide range of habitats.^4^ *Culex pipiens* uses different water sources, including freshwater sources, such as ponds, semi-permanent waters, larger pools with vegetation, rice fields, areas along river edges with minimal water flow, and inundation areas.^4^^,^^48^ These habitats offer suitable breeding sites, typically with stagnant waters and aquatic vegetation, that support a diverse array of avian and mammalian hosts, providing ample opportunities for mosquitoes to feed on birds and acquire avian malaria parasites.^49^ The environmental requirements may explain, at least in part, the negative relationships between *Plasmodium* prevalence and the distance to different water reservoirs as parasite transmission may increase with the abundance of areas suitable for the main vectors in the area. Our results agree with previous studies reporting an increased probability of *Plasmodium* infection in the proximity of freshwater, ponds, and slow rivers.^21^ Interestingly, Illera et al.^28^ found a negative association between *Plasmodium* prevalence and richness in birds and the distance to water sources in a close Mediterranean mountain (i.e., Sierra Nevada) locality. Authors from this study suggested that water availability may represent a limiting factor for vector development in this environment, as may be the case of our study. Therefore, the link between the distance to water sources and parasite infections in birds may be explained by the effects of water sources providing favorable conditions for breeding and development of insect vectors, which may also determine the prevalence of infection in mosquitoes.

We found positive relationships between parasite prevalence in mosquito pools and the distance to both salt marshlands and rivers, as well as between parasite richness and the distance to freshwater stretches. Salt marshes might constitute less favorable habitats for *Cx. pipiens*,^50^ thereby resulting in a reduction in parasite prevalence. For instance, marshland environments may lack certain characteristics or resources crucial for sustaining *Cx. pipiens* populations, such as suitable breeding sites or optimal conditions for the survival and reproduction of mosquitoes.^4^ By contrast, *Ae. caspius* is especially common in salt marshes;^20^ however, this species has a higher preference to bite on mammals than in birds, potentially reducing the prevalence of parasites found in mosquitoes. In fact, a previous study identified a negative association between the seroprevalence of the mosquito-borne WNV in wild birds and the captures of *Ae. caspius*,^22^ a pattern which could also be found in ornithophilic mosquitoes. In addition, we found a positive correlation between *Plasmodium* prevalence in mosquitoes and the distance to the rivers which could be due to the presence of aquatic predators of mosquito larvae (e.g., fish) reducing mosquito populations.^51^ The spatial distribution of these mosquito predators in the proximity to rivers can indeed naturally avoid mosquito breeding, influencing the overall prevalence of mosquito-borne parasites in the surrounding areas. Finally, richness of parasite lineages in *Cx. pipiens* increased farther from freshwater stretches. These areas are likely to harbor a greater diversity of mosquito species,^4^ each with varying competencies for hosting and transmitting different *Plasmodium* lineages.^48^ Indeed, in freshwater communities, mosquito larvae interact with both larval competitors and shared predators,^52^ potentially diluting *Plasmodium* lineages circulation due to increased competition among mosquito species.

Variations in the number of parasite lineages circulating among different habitats have been previously documented in birds. For example, Jiménez-Peñuela et al. (2021) reported a higher richness of *Haemoproteus* lineages in house sparrows from rural habitats compared to natural areas, albeit in a single year of their study. However, no differences were found for *Plasmodium*. Conversely, our investigation reveals a contrasting pattern, with greater *Plasmodium* lineage richness in mosquitoes from natural areas compared to rural and urban environments, which harbor a subset of these lineages. These disparities among studies underscore the intricate interplay of factors influencing parasite lineage distribution, including the specific parasite species under investigation (i.e., *Plasmodium*, which is primarily transmitted by mosquitoes, as opposed to *Haemoproteus*^1^), potential interannual variations in parasite dynamics, and the role of bird species in filtering specific lineages. Furthermore, the varying host specificity of avian *Plasmodium* lineages^53^^,^^54^ and the known host restrictions of certain parasite morphospecies^55^ contribute to the complexity of these habitat-driven prevalence patterns.

The composition and structure of the avian community within different habitats could influence the distribution and prevalence of *Plasmodium* lineages in mosquitoes. Patterns found here could also be explained based on different compositions of hosts, parasites, and vectors between areas. For example, differences in the abundance of *Cx. pipiens* mosquitoes were found between rural and natural areas in southern Spain, with higher captures in natural habitats.^20^ In addition, the ability of parasite lineages to circulate in a particular area may be driven by the vector communities present, based on the differential capacity of parasites to infect different species. Indeed, the negative relationship found between parasite prevalence and the total abundance of mosquitoes can suggest the higher abundance of non-competent vectors in the community, such as the two most trapped species *Cx. theileri* and *Ae. caspius*,^20^ that could reduce the overall parasitic load circulation.

*Plasmodium* are considered generalist parasites being able to infect birds of different species,^56^^,^^57^ but there are clear differences between parasite lineages and species.^58^ Although different genera of mosquitoes can harbor the same *Plasmodium* lineages and potentially be involved in their transmission,^9^^,^^21^^,^^59^ experimental infection studies have identified interspecific differences in the vector competence of mosquitoes for the transmission of avian *Plasmodium*.^3^ In our study, natural areas were defined as those with a comparatively higher conservation status, while livestock dominate in rural areas. These differences may modify the structure and composition of the bird and vector communities, finally affecting the parasite lineages infecting mosquitoes. In this respect, the positive correlation between mosquito and parasite richness in the areas suggests the possibility that the presence of different species of mosquitoes may provide further opportunities for the local transmission of different parasite lineages. For example, the presence of other competent vectors for *Plasmodium* parasites also present in the area, such as *Cx. perexiguus* or *Cx. modestus*,^2^ may favor the local circulation of parasites in the area finally reaching *Cx. pipiens* mosquitoes.

### Limitations of the study

While our research provides valuable insights into factors affecting the patterns of infection by avian *Plasmodium* in mosquitoes, certain limitations merit consideration. Firstly, our study primarily focuses on *Cx. pipiens*, a key vector species of avian *Plasmodium*, but other vectors may contribute to the overall transmission dynamics of these parasites in the area.^1^^,^^4^ Furthermore, our study is geographically confined to southern Spain, and caution is needed when generalizing findings to other regions with different ecological characteristics. Lastly, we sampled mosquitoes during a single year, but longitudinal studies would better capture temporal variations of parasite dynamics in the area. Future studies may contribute to solve these limitations improving our understanding of avian *Plasmodium* transmission dynamics.

### Concluding remarks

We present evidence that habitat type, water source, and mosquito community composition play an important role in the transmission patterns of avian malaria parasites in *Cx. pipiens* mosquitoes. This is especially relevant because most studies conducted until now to identify the impact of landscape on the dynamics of transmission of *Plasmodium* parasites argue that the observed patterns may be explained by their impacts on vectors, but in most of the cases these relationships remain unexplored. Mosquitoes, due to their shorter lifespan, faster life cycle, and reduced mobility compared to avian hosts, can provide a more immediate and detectable reflection of environmental effects determining the prevalence and richness of avian malaria parasites, especially when transmitted by competent insect vectors like *Cx. pipiens*.

## STAR★Methods

### Key resources table

**Table:** 

REAGENT or RESOURCE	SOURCE	IDENTIFIER
**Biological samples**		

*Culex pipiens* mosquitoes	DNA	N/A

**Deposited data**		

Sequencing files	GenBank database	OR750857

### Resource availability

#### Lead contact

Further information and requests for resources and reagents should be directed to and will be fulfilled by the lead contact, Dr. Martina Ferraguti (mferraguti@ebd.csic.es).

#### Materials availability

This work did not generate new unique reagents.

#### Data and code availability

Data reported in this paper will be shared by the lead contact upon request.

• This paper does not report original code.
• Sequencing data files have been deposited at GenBank database and are publicly available as of the date of publication. Accession numbers are listed in the Table S1 and in the key resources table.
• Any additional information required to reanalyse the data reported in this paper is available from the lead contact upon request.

### Experimental model and study participant details

#### Ethics statement

Mosquito trapping was carried out with all the necessary permits from Consejería de Medio Ambiente, and Consejería de Agricultura, Pesca y Desarrollo Rural (Junta de Andalucía). Entomological surveys on private land and in private residential areas was conducted with all the necessary permits and consent, and in the presence of owners. This study did not affect any endangered or protected species.

### Method details

#### Mosquito sampling, identification and parasite screening

Mosquitoes were collected during 2013 at 45 distinct localities in the provinces of Cadiz (n = 15), Huelva (n = 15) and Seville (n = 15) in Andalusia, southern Spain. The study area is characterized by a Mediterranean climate, with winter rainfall and an extended dry period in the summer. To ensure a comprehensive representation of the habitat types in the region, the sampling sites were organized into triplets. Each triplet encompassed one locality in a natural habitat, another in a rural habitat, and a third in an urban habitat. The selection of these three habitat types was based on the visual inspection of the areas. Urban habitats were characterized by higher human population densities compared to the other two types. Rural habitats exhibited a greater density of livestock in comparison to urban and natural habitats. Natural habitats, in contrast, had lower densities of both humans and livestock while maintaining a generally well-preserved environment (see Ferraguti et al.^20^ for further details on habitat characterization).

The mosquitoes were captured in the framework of the study carried out by Ferraguti et al.^20^ Briefly, from April to December 2013, insects were trapped using BG-sentinel traps baited with dry ice as a source of CO_2_ operating for 24 h. Every 45 days, we sampled mosquitoes in each locality using three traps per locality with a mean distance between traps of 119 m (range 20–636 m). Female mosquitoes were preserved in dry ice and transported to the laboratory for their identification at species level using available morphological keys,^4^ including the *MosKeyTool* software (https://www.medilabsecure.com/moskeytool).

*Culex pipiens* females without any evidence of a recent blood meal in their abdomen were grouped in pools containing 1–50 individuals collected on the same day and locality of capture. Each mosquito pool was homogenized in 500–700 mL of minimal essential medium (MEM solution) supplemented with 200 U/ml of antimicrobial drugs (penicillin/streptomycin) and 10% fetal bovine serum, and then stored at −80°C for further analysis. The genomic DNA of mosquito pools was extracted using a QIAamp Viral RNA kit (Qiagen, Germany) according to the manufacturer’s recommendations. The presence and identity of *Plasmodium* parasites were identified following Hellgren et al..^60^ Amplicons were sequenced in the Macrogen sequencing service (Macrogen Inc., Madrid) and parasite lineages were determined by BLAST comparison of the sequences with those deposited in GenBank and MalAvi.

To estimate the prevalence of blood parasites in mosquitoes, *EpiTools* software (AusVet Animal Health Services, Australia; https://epitools.ausvet.com.au/) was used. This procedure considers differences in pool size (i.e., number of mosquitoes included in each mosquito pool), the number of mosquito pools analyzed and the number of positive mosquito pools per sampling locality. We assumed a 100% sensitivity and specificity in the analyses. Parasite richness was estimated using rarefaction curves to standardize parasite richness estimates accounting for variations in the number of mosquito pools collected across different localities.^61^^,^^62^

#### Assessment of water sources through remote sensing

Spatial distribution of the main water sources was obtained from cartography accessible at http://www.juntadeandalucia.es/institutodeestadisticaycartografia/DERA/using ArcGIS v10.2.1 (ESRI, Redland). Regarding hydrological variables, we assessed the mean distance, in meters, of three traps at each of the 45 localities to different water sources. These sources included the distance to i) the nearest river courses, ii) any salt marsh patches, iii) stretches of freshwater (e.g., all inland water bodies, considering both natural and artificial), and iv) existing water reservoirs (e.g., basins and deposits). Additionally, we calculated v) the shortest distance to any water source by aggregating raster information from the aforementioned variables.

### Quantification and statistical analysis

We independently analyzed the *Plasmodium* prevalence and richness of lineages, as they reflect different aspects of the infectious status of mosquitoes. The effects of i) habitat type (categorical variable: natural, rural or urban); ii) distances to the different water sources including the shortest distance to river, salt marsh, stretch of freshwater, water reservoirs and any type of water source (all continuous variables); iii) total abundance of mosquitoes, assessed as the cumulative count of female mosquitoes belonging to each captured species per locality; iv) mosquito richness, estimated by the richness of lineages of *Plasmodium* estimated from rarefaction curves^61^^,^^62^; and v) mosquito diversity, measured by estimating the evenness from the Shannon’s equitability index per locality,^63^ were tested by fitting Linear Mixed-Effects Models (LMMs) with a Gaussian distribution. A backward stepwise selection procedure was applied by removing either the variable or the interaction with the highest p values. This procedure was used until obtaining a simplified model including only variables with p ≤ 0.10. In the case of interactions with p ≤ 0.10, both variables affected were retained in the final model. Data for mosquito community abundance and species composition were taken from Ferraguti et al.^20^ where detailed information of the mosquito species sampled is provided. Overall, we collected 13 different mosquito species, including *Culex theileri* (282 891 ind.), *Aedes caspius* (21 155), *Culex pipiens* (19 268), *Culex perexiguus* (5,939), *Anopheles atroparvus* (5,387), *Culiseta annulata* (2,514), *Aedes detritus* (1,495), *Culex modestus* (1,237) and *Culiseta longiareolata* (476) as the most common species sampled. Total abundance of mosquitoes, and all the distances to water sources were log-transformed to normalize its distribution and stabilize the variance and to deal with differences of several orders of magnitude between sampling sites. Province and triplet nested into province, were included in all models as random factors to account for the geographical stratification of the sampling design. Collinearity between variables included in the models were assessed according to the variance inflation factor.^64^ The marginal and conditional variances explained (*R*^*2*^) by both fixed and random factors were calculated following Nakagawa and Schielzeth,^65^ thus providing an absolute value for the variance explained by the models.

To determine and visually represent the associations between the *Plasmodium* lineage compositions in the three distinct habitat types, a heatmap was constructed using the function *heatmap.2* from the *ggplot2* package, depicting the frequency of each parasite lineage at each sampling locality. All analyses were developed in R version 2.14.2 (R Development Core Team 2005).

### Additional resources

Any additional information required to reanalyse the data reported in this paper is available from the lead contact upon request or in the Table S1.
