# Supplementary material for: Landscape and mosquito community impact the avian Plasmodium infection in Culex pipiens
Source: iScience. 2024 Feb 10;27(3):109194. doi: 10.1016/j.isci.2024.109194 (PMC10906513; doi:10.1016/j.isci.2024.109194)
Supplement: Document S1. Table S1 [file mmc1.pdf]

iScience, Volume *27*

Supplemental information

Landscape and mosquito community impact the avian *Plasmodium* infection in *Culex pipiens*

Martina Ferraguti, Josué Martínez-de la Puente, Santiago Ruiz, Ramón C. Soriguer, and Jordi Figuerola

Appendix A - Supplementary data

Landscape and mosquito community impact on avian *Plasmodium* infection in *Culex pipiens*

By: Martina Ferraguti, Josué Martínez-de la Puente, Santiago Ruiz, Ramón C. Soriguer, Jordi Figuerola

Table S1. *Plasmodium* lineages and associated morphological species (when available) found in this study.

***Plasmodium* lineage**	**Morphospecies**	**GenBank accession number**	**Nº of *Cx. pipiens* infected pools**
COLL1	*Plasmodium sp.*	AY831747	7
COTCOT01	*Plasmodium sp.*	JN164708	1
CXPER01[1]	*Plasmodium sp.*	HM179147	51
CXPIP23[2]	*Plasmodium sp.*	JF411405	4
DELURB5	*Plasmodium sp.*	EU154347	12
GRW11	*Plasmodium relictum*	AY831748	3
LINN1	*Plasmodium matutinum*	DQ847270	26
CXPIP35	*Plasmodium sp.*	OR750857	1
PADOM01	*Plasmodium sp.*	DQ058611	1
SGS1	*Plasmodium relictum*	AF495571	22
SGS2	*Plasmodium sp.*	JN661991	1
SYAT05	*Plasmodium vaughani*	DQ847271	104
YWT4[3]	*Plasmodium sp.*	DQ368395	3

[1] Accession number refers to the lineage synonyms DONANA10 described in MalAvi (version 2.5.8).
[2] Accession number refers to the lineage synonyms DONANA09 described in MalAvi (version 2.5.8).
[3] Accession number refers to the lineage synonyms DONANA06 described in MalAvi ((version 2.5.8).
